# Using hospital readmission rates to track the quality of care in public hospitals in Singapore

**DOI:** 10.1186/1472-6963-11-S1-A16

**Published:** 2011-10-19

**Authors:** E Lim, N Matthew, W Mok, S Chowdhury, D Lee

**Affiliations:** 1 Healthcare Performance Group, Ministry of Health Singapore, Singapore 169854

## Research objectives

Singapore introduced Casemix-based financing for inpatient and day-surgery cases in the public sector in 1999. The application of Casemix has since been extended beyond financing to fields such as benchmarking, clinical quality and utilization review. Casemix data has been invaluable in enabling the tracking and better understanding of quality of care of healthcare providers, as well as providing a view to better managing them.

In this paper, we discuss the use of Casemix data based on a recent study of hospital readmission rates. The study subsequently led to the incorporation of this indicator into the Ministry of Health’s Scorecard for Acute Hospitals.

## Methods

Hospital administrative data of inpatients admitted to public hospitals in Singapore during 2006–2010 were analyzed. 30-day readmission rates were calculated after excluding ‘transfers-out’, ‘in-hospital deaths’, and cases with certain underlying conditions that might potentially affect the risk of readmission (for example, cancer, HIV, trauma). The rates were further adjusted for patients’ Casemix using multivariate logistic linear regression modeling to ensure like-for-like comparisons when comparing hospitals and evaluating trends over time. Factors for adjustment included age, gender, Charlson comorbidity index, and past hospitalization.

Readmission rates were analysed at the ‘All cause’ level as well as at the ‘Condition-specific’ level; i.e., for seven selected conditions: asthma, AMI, CHF, COPD, diabetes, pneumonia, and stroke.

## Results

In 2010 the crude ‘All cause’ 30-day readmission rate was 11.6%. Of those readmitted, the admission of 27.3% was due to the same principal diagnosis, and 83.6% returned to the same index hospital. It was found that rates were higher with increasing age. Also identified as the most significant risk factors affecting readmissions were hospitalization in past year, the Charlson comorbidity index, and principal diagnoses of index episodes. In those aged 65 years and older, the readmission rate in Singapore was 19.0%, slightly lower than in the United States (19.6%).

The study also highlighted differences in readmission rates between hospitals, indicating a likely variation in quality of care. This was present at both the ‘All cause’ and the ‘Condition-specific’ levels.

## Conclusion

Readmission rate was assessed as to its validity as an effective ‘Big Dot’ measure for inclusion in the Ministry of Health’s performance measurement and quality improvement framework for acute hospitals. The findings for this indicator have since been shared with the hospitals which subsequently worked out targeted solutions to close performance gaps, with the ultimate goal of raising the quality of patient care. The indicators will continue to be reviewed regularly, and the performance of hospitals will be tracked to monitor improvement over time.
